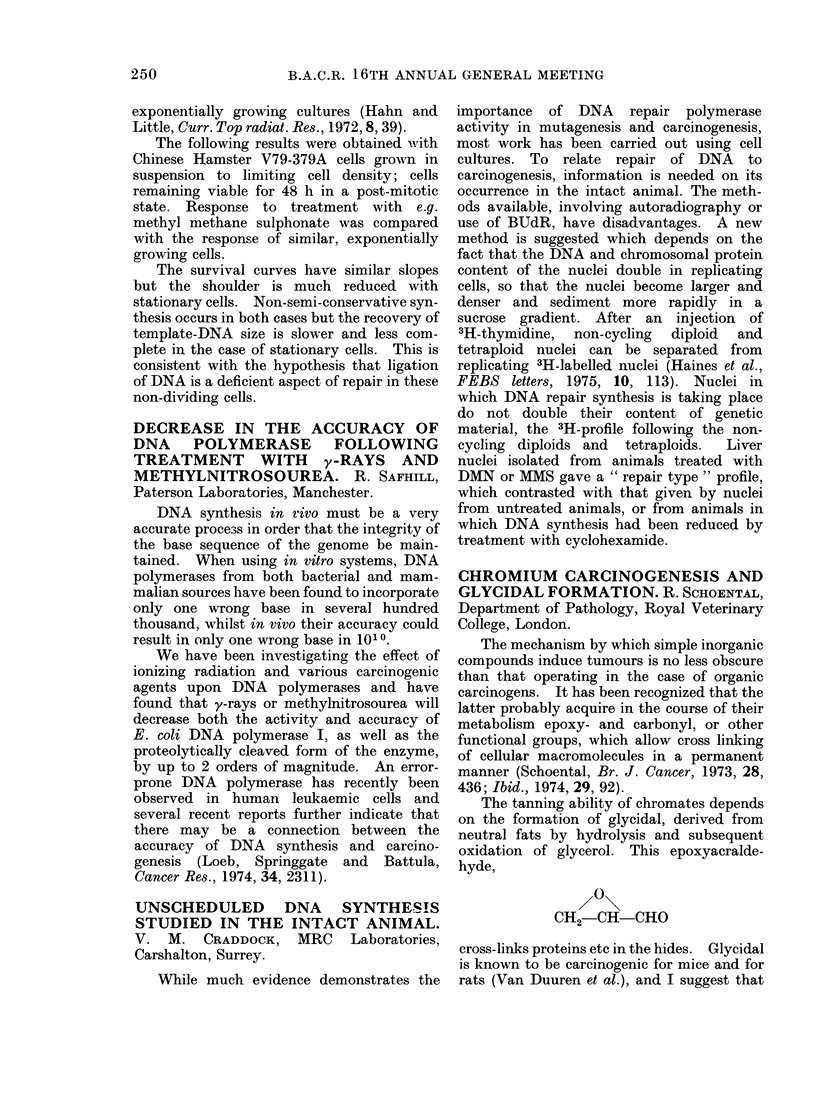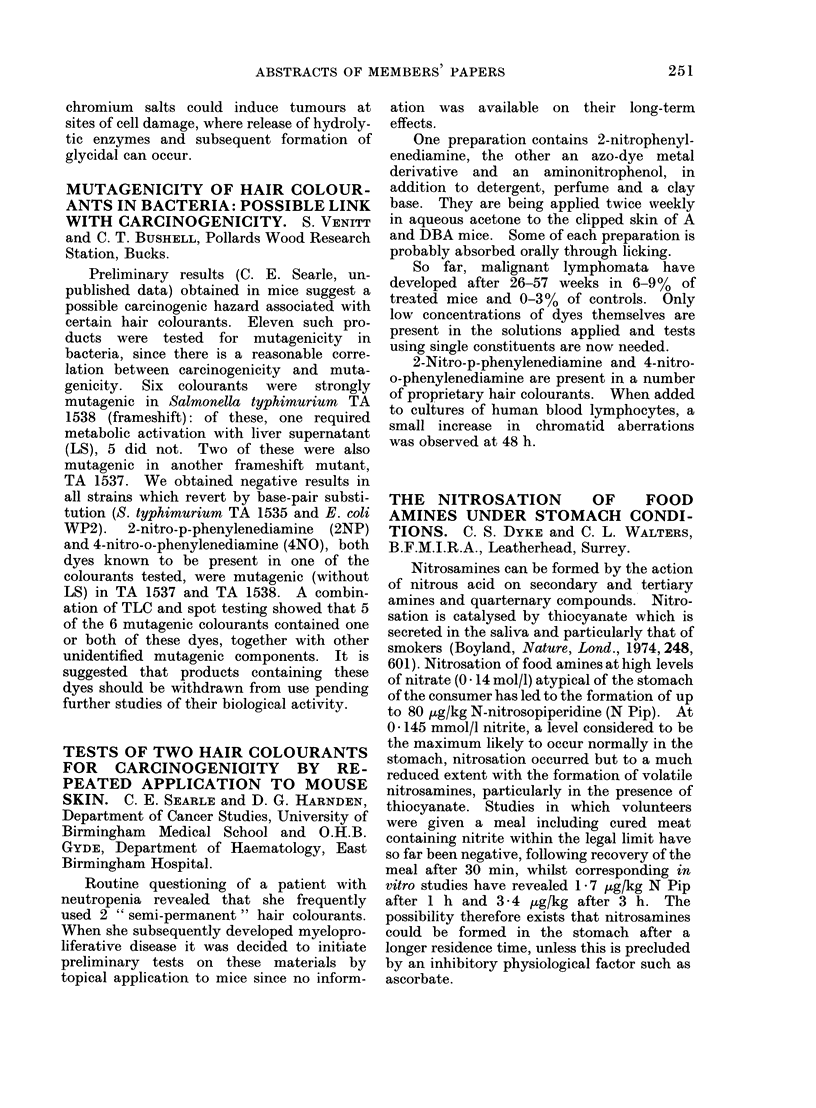# Proceedings: Chromium carcinogenesis and glycidal formation.

**DOI:** 10.1038/bjc.1975.190

**Published:** 1975-08

**Authors:** R. Schoental


**CHROMIUM CARCINOGENESIS AND GLYCIDAL FORMATION.** R. SCHOENTAL, Department of Pathology, Royal Veterinary College, London.

The mechanism by which simple inorganic compounds induce tumours is no less obscure than that operating in the case of organic carcinogens. It has been recognized that the latter probably acquire in the course of their metabolism epoxy- and carbonyl, or other functional groups, which allow cross linking of cellular macromolecules in a permanent manner (Schoental, *Br. J. Cancer*, 1973, **28**, 436; *Ibid.*, 1974, **29**, 92).

The tanning ability of chromates depends on the formation of glycidal, derived from neutral fats by hydrolysis and subsequent oxidation of glycerol. This epoxyacraldehyde, cross-links proteins etc in the hides. Glycidal is known to be carcinogenic for mice and for rats (Van Duuren *et al.*), and I suggest that chromium salts could induce tumours at sites of cell damage, where release of hydrolytic enzymes and subsequent formation of glycidal can occur.